# Temperature of gas delivered from ventilators

**DOI:** 10.1186/2052-0492-1-6

**Published:** 2013-11-08

**Authors:** Yusuke Chikata, Mutsuo Onodera, Hideaki Imanaka, Masaji Nishimura

**Affiliations:** Emergency and Critical Care Medicine, The University of Tokushima Graduate School, 3-18-15 Kuramoto, Tokushima City, 770-8503 Japan; Medical Equipment Center, Tokushima University Hospital, 2-50-1 Kuramoto, Tokushima City, 770-8503 Japan; Emergency and Disaster Medicine, Tokushima University Hospital, 2-50-1 Kuramoto, Tokushima City, 770-8503 Japan

## Abstract

**Background:**

Although heated humidifiers (HHs) are the most efficient humidifying device for mechanical ventilation, some HHs do not provide sufficient humidification when the inlet temperature to the water chamber is high. Because portable and home-care ventilators use turbines, blowers, pistons, or compressors to inhale in ambient air, they may have higher gas temperature than ventilators with piping systems. We carried out a bench study to investigate the temperature of gas delivered from portable and home-care ventilators, including the effects of distance from ventilator outlet, fraction of inspiratory oxygen (F_I_O_2_), and minute volume (MV).

**Methods:**

We evaluated five ventilators equipped with turbine, blower, piston, or compressor system. Ambient air temperature was adjusted to 24°C ± 0.5°C, and ventilation was set at F_I_O_2_ 0.21, 0.6, and 1.0, at MV 5 and 10 L/min. We analyzed gas temperature at 0, 40, 80, and 120 cm from ventilator outlet and altered ventilator settings.

**Results:**

While temperature varied according to ventilators, the outlet gas temperature of ventilators became stable after, at the most, 5 h. Gas temperature was 34.3°C ± 3.9°C at the ventilator outlet, 29.5°C ± 2.2°C after 40 cm, 25.4°C ± 1.2°C after 80 cm and 25.1°C ± 1.2°C after 120 cm (P < 0.01). F_I_O_2_ and MV did not affect gas temperature.

**Conclusion:**

Gas delivered from portable and home-care ventilator was not too hot to induce heated humidifier malfunctioning. Gas soon declined when passing through the limb.

## Background

During unaided spontaneous breathing (SB), inspiratory gases are usually heated and humidified in the nasal cavity and pharynx. By the time the second bronchial bifurcation is reached, inspired gas temperature is 37°C, and absolute humidity is 44 mg/L [1]. When patients are mechanically ventilated, however, the anatomy that provides this natural conditioning is bypassed, and it is necessary to warm and humidify medical gases. Heated humidifiers (HHs) and heat-and-moisture exchangers (HMEs) are commonly used humidifying devices. While HMEs are easy to use and have growing popularity, HHs are indicated for patients with air leakage or acute respiratory distress syndrome, especially with hypercapnia [2–4].

Sophisticated HHs monitor temperature at the water-chamber outlet and at the end of the inspiratory circuit. Feedback circuits control electric power to the water-chamber heating plate and the heating wire inside the inspiratory circuit, thus maintaining the temperature and humidity of inspiratory gas at 37°C and ensuring 100% relative humidity at the proximal end of the endotracheal tube. This control system may have untoward effects, for example, gas delivered from the ventilator is too hot, the higher temperature detected at the water-chamber outlet causes the HH to decrease electric power to the heating plate resulting in less vapors in the inspiratory gas [5].

As reported by Austin et al., the gas temperature of ventilator-outlet, warmed by heat generated by the ventilator, may rise as high as 46.7°C [6]. The hottest ventilator was an ultracompact portable model, which incorporated a turbine to inhale ambient air. Expecting to see considerable improvement in power efficiency during the decade and more since their study, and a more recent model of the design, we carried out a bench study to investigate ventilator-outlet temperature, and the effects of distance from ventilator-outlet to HH and of fraction of inspiratory oxygen (F_I_O_2_) and minute volume (MV) on temperature.

## Methods

**Figure 1:**
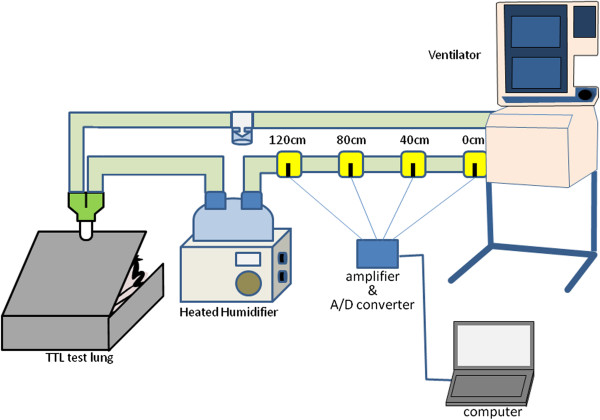
**Experimental set-up.** Starting at the ventilator outlet, in the tube from ventilator to heated humidifier, to monitor gas temperature at each location, single thermistors were placed at 40 cm intervals. The respiratory circuit was completed to return expired gas from the test lung to the ventilator. Since the only concern was to measure gas delivered from the ventilator, the heated humidifier was not turned on.

We assessed three portable ventilators, Newport HT70 (Newport Medical Instruments; Mansfield, MA, USA), LTV1200 (CareFusion, Minneapolis, MN, USA), iVent (GE Healthcare; Wisconsin, WI, USA), one non-invasive positive pressure ventilator, BiPAP Vision (Philips Respironics; Carlsbad, CA, USA), and one ICU ventilator with and without an air compressor, Puritan Bennett 840 (Covidien; Pleasanton, CA, USA) (Table 1). In the inspiratory limb, single thermistors were placed at four locations: one at the ventilator inspiratory gas outlet, and one at every 40 cm downstream (Figure 1). A low compliance ventilator circuit (DAR breathing system tube) connected the ventilator outlet to the HH water-chamber. Downstream from the humidification chamber, the original ventilator tubing that came with each ventilator was used. We used the MR730 HH (Fisher & Paykel, Auckland, New Zealand) because it is the standard HH in our ICU. A test lung (TTL model 1601, Michigan Instruments, Grand Rapids, MI, USA) was ventilated using assist/control with tidal volume (V_T_) set at 500 mL, frequency at 10 and 20 breaths/min, inspiratory time at 1.0 s and F_I_O_2_ at 0.21, 0.6, and 1.0. Compliance of the test lung was 0.05 L/cmH_2_O. To obtain accurate measurements solely of the outlet gas temperature, the HHs were not turned on during study. All experiments were performed in an air-conditioned room in which ambient temperature was monitored with another thermistor (Moiscope, Senko Medical, Tokyo, Japan).

**Table 1:** **Tested ventilators**

Ventilator	Manufacture	Type of supply system	Type of flow sensor
LTV1200	Philips Respironics	Turbine	Differential pressure
HT70	Newport MEDICAL	Piston	None
iVent	GE Healthcare	Blower, turbine	Differential pressure
BiPAP Vision	Philips Respironics	Blower	Differential pressure
PB840 + compressor	Covidien	Compressor	Hot wire flow sensor
			(190°C ~ 200°C)
PB840	Covidien	Piping	Hot wire flow sensor
			(190°C ~ 200°C)

All four thermistors (NT-1000, San-You Technology Ltd, Saitama, Japan) in the ventilator circuit were two-point calibrated using a cooler/heater water source (HHC-51, Senko Medical, Tokyo, Japan). As a preliminary, each ventilator was left to run until the gas temperature of ventilator outlet became constant. We ascertained that this was achieved by all the tested ventilators by about 5 h. After confirming it, we recorded gas temperature at the four sites for 1 min. After recording the gas temperature following each change of ventilator setting, we waited for at least 30 min before collecting new data. All signals from the thermistors were processed through an analog/digital converter and saved on a computer at the sampling rate of 50 Hz/channel using data acquisition software (WinDaq, Dataq Instruments, Akron, OH, USA). Analysis of variance was performed using repeated measures ANOVA. All statistical testes were two-sided and a P value<0.01 was considered statistically significant. All statistical analysis was performed using commercial software (SPSS 11.01, SPSS, Chicago, IL, USA). Results were expressed as mean ± SD.

## Results

**Figure 2:**
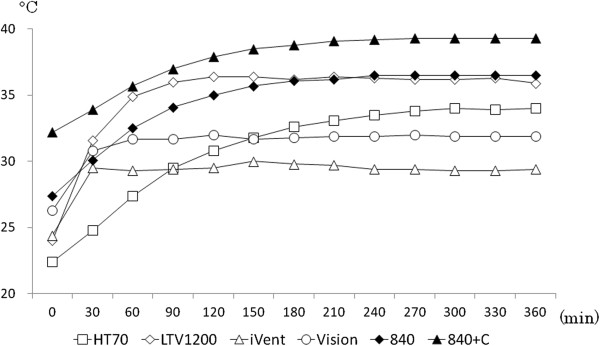
**Changes in delivered gas temperature over time.** These readings are for gas temperature recorded at the ventilator outlet. The range of temperature of gas which differed as delivered by different ventilators was as wide as 10°C.

Throughout each test, room temperature was 24.0°C ± 0.5°C. After the ventilators started running, for up to 5 h, outlet gas temperature gradually increased to a stable level (Figure 2). Depending on the ventilator, early gas temperature varied from 22.4°C to 32.2°C (26.1°C ± 3.5°C) among the ventilators, and then gradually rose within 5 hrs or less to a stable temperature in the range of 29.4°C to 39.3°C (34.5°C ± 3.5°C). Consequently, we evaluated the effects of setting changes after gas temperature was stable for all ventilators, that is, after 6 h of operation.

**Figure 3:**
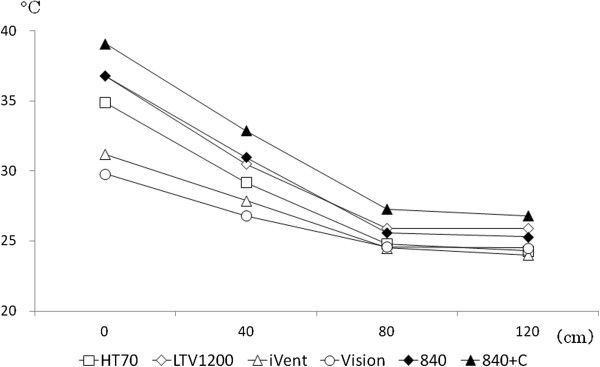
**Effect of distance from outlet on temperature of gas delivered from the ventilators.** For each ventilator, by the time the gas had traveled 40 cm from the outlet, gas temperature fell less than 35°C.

In all the ventilators, gas temperature fell to below 35°C before the gas reached 40 cm from the outlet, and at 80 cm, gas temperature was below 30°C (*P* < 0.01) (Figure 3). No decrease in gas temperature was apparent after 80 cm. Gas temperature was not affected by changes in F_I_O_2_ or in MV. In both, differences within ±5% are not considered significant.

## Discussion

In this study evaluating the delivered gas temperature of various ventilators, we found variations as much as 10°C in the outlet gas temperature of different ventilators. Outlet gas temperature increased gradually after the ventilators began operating, and it took one of the ventilators as long as 5 h to provide gas at a stable temperature. Gas temperature decreased rapidly as the gas traveled up to 80 cm from the ventilator outlet. Neither F_I_O_2_ nor MV had any apparent influence on gas temperature.

Our results show that the gas temperature delivered from the ventilators was not so high as to cause this type of HH malfunctioning [5].

The gas temperature from ventilator is dependent on both ambient temperature and absorption of heat generated by the friction between the gas and internal pneumatic circuits and electrical circuit during the working of the ventilator. Portable ventilators usually inhale ambient air and control F_I_O_2_, so high ambient air temperature would result in correspondingly high gas temperature. Heat release may also influence gas temperature, and it could be poor with the ultracompact ventilator. All the ventilators tested performed adequately in our tests, which were carried out in an air-conditioned room with ambient temperature at 24.0°C ± 0.5°C. In an ambient temperature of 22.9°C ± 0.2°C, Lellouche et al. found gas temperature was 29.8°C ± 1.0°C, which did not lead to HH malfunction [5]. When the ambient temperature was higher (29.0 ± 0.2°C), however gas temperature increased to 35.8°C ± 1.5°C [5].

Since the temperature of the working ventilator will always be greater than the ambient temperature and, in addition, heat release is more efficient when ambient temperature decreases, it remains unclear which factor influences gas temperature the most in different circumstances. We found the highest gas temperature with the PB840 supplied with the compressor, which was not surprising because the input gas temperature was greater than the ambient air temperature. Gas temperature was also relatively high with the turbine-equipped, ultracompact LTV1200. When we changed F_I_O_2_, it had no discernible influence on the medical gas temperature. Only when the temperature of oxygen gas and ambient air differs greatly, would the delivered gas temperature be affected by F_I_O_2_. This type of effect is illustrated by the delivered gas temperature from the PB840 supplied by compressor.

We found the temperature difference between MV of 5 L/min and 10 L/min was less than 5%, but we must also point out that MV was changed only by increasing the respiratory rate, from 10 to 20 breaths/min. Gas flow *per se* was not altered to modulate MV.

Gas travel distance from the ventilator outlet greatly influenced gas temperature. In a limb not warmed by a heating wire, lower-temperature air surrounding the tube easily carries away heat. Chikata et al. reported that the temperature of gas passing through a tube as short as 10 cm, if a heating wire is not used, can be greatly affected by ambient temperature [7]. In the present study, gas temperature decreased as the gas traveled up to 80 cm from the ventilator outlet. How much it decreases is dependent on gas flow and the ambient air. Minute ventilation of our settings was 5 and 10 L/min, and gas temperature did not differ between them. Gas flow might hardly influence gas temperature. Ambient temperature was the most important factor, and we should be careful when we use it under high ambient temperature, such as radiology lab. HME is suitable under transporting or under high ambient temperature.

We investigated only one ventilator of each brand. As Lellouche et al. investigated three different days and obtained different results, gas temperature was not always same. We did not repeat our measurement in the present study, and we cannot deny the possibility of this issue.

## Conclusion

In conclusion, in none of the ventilators gas temperature was high enough to cause HH malfunction. Even when gas temperature was relatively high at the ventilator outlet, it soon decreased to close to ambient temperature after traveling just 80 cm.
