# Correction: Temperature of gas delivered from ventilators

**DOI:** 10.1186/2052-0492-1-16

**Published:** 2013-12-20

**Authors:** Yusuke Chikata, Masayo Izawa, Hideaki Imanaka, Nao Okuda, Masaji Nishimura

**Affiliations:** 1 Emergency and Critical Care Medicine, The University of Tokushima Graduate School, 3-18-15 Kuramoto, Tokushima City 770-8503, Japan; 2 Medical Equipment Center, Tokushima University Hospital, 2-50-1 Kuramoto, Tokushima City 770-8503, Japan; 3 Emergency and Disaster Medicine, Tokushima University Hospital, 2-50-1 Kuramoto, Tokushima City 770-8503, Japan

## Correction

After publication of this work [1], we noted that we inadvertently provided an incorrect author list. The correct list of authors is now included and the Authors' contributions and Competing interests sections modified accordingly.

## Competing interests

The authors declare that they have no competing interests.

## Authors’ contributions

YC and MN conceived the study. YC and MN participated in the design of the study. YC, MI, HI, NO, and MN participated in data interpretation, and drafted the manuscript. All authors read and approved the final manuscript.

## References

[B1] Chikata Y Onodera M Imanaka H Nishimura M Temperature of gas delivered from ventilators J Intensive Care 2013 1 6 10.1186/2052-0492-1-6 25705400 PMC4336268

